# Non-invasive estimation of coronary resistance and compliance: Prospective diagnostic study vs. angiography

**DOI:** 10.1016/j.ahjo.2026.100763

**Published:** 2026-03-18

**Authors:** Byoung-Kwon Lee, Seog-San Hyeon, YouSik Hong, Dae-Woong Choi, Sang-Suk Lee

**Affiliations:** a Cardiovascular Center, Department of Internal Medicine, Gangnam Severance Hospital, College of Medicine, Yonsei University, Seoul, 06273, Republic of Korea; b Technology Research Institute, IRUMEDI Co., Ltd., Goyang, Gyeonggi-do, 10442, Republic of Korea; c Department of Software, College of Engineering, Sangji University, Wonju, Gangwon-do, 26339, Republic of Korea

**Keywords:** Coronary artery disease (CAD), Resistance, Compliance, Atherosclerosis, Sensitivity, Specificity, Coronyzer (KH-3000)

## Abstract

**Background:**

Early identification of coronary artery disease (CAD) remains challenging, particularly in patients who cannot undergo exercise-based or contrast-dependent diagnostic testing. We evaluated a non-invasive pulse wave–based device, Coronyzer (KH-3000), designed to derive resistance and compliance indices for early screening of CAD.

**Methods:**

A prospective diagnostic accuracy study was conducted in 94 patients with suspected angina who underwent coronary angiography (CAG). Significant CAD was defined as ≥50% diameter stenosis. Diagnostic performance was assessed using pre-specified thresholds for resistance (R) and compliance (C). An independent retrospective validation study was performed in 136 patients who underwent CAG and computed tomography coronary angiography (CTCA). Diagnostic performance was evaluated using predefined OR and AND decision rules (*R* > 1.24 and/or C < 0.8).

**Results:**

In the prospective cohort, sensitivity and specificity were 81% and 89%, respectively. In the validation cohort, the OR rule demonstrated high sensitivity (0.77) with lower specificity (0.41), whereas the AND rule showed lower sensitivity (0.53) but high specificity (0.78). Receiver operating characteristic (ROC) analysis demonstrated moderate overall diagnostic accuracy (area under the curve (AUC) = 0.67).

**Conclusions:**

Coronyzer demonstrated clinically meaningful diagnostic performance as a non-invasive screening and risk stratification tool for coronary artery disease. By avoiding radiation exposure, contrast agents, and exercise requirements, the device may support early clinical triage and referral for further diagnostic evaluation, particularly in patient populations for whom conventional testing is limited.

## Highlights

• The pulse-wave device estimates coronary resistance/compliance w/o exercise/contrast.
• The prospective CAG study showed 81% sensitivity and 89% specificity for ≥50% stenosis.
• Validation: OR rule improved sensitivity; AND rule improved specificity.
• Prespecified *R* > 1.24 and C < 0.8 thresholds enabled two complementary decision rules.
• Overall discrimination was moderate (AUC 0.67) in the validation cohort.

## 1 Introduction

Coronary artery disease (CAD) is a leading cause of mortality worldwide. While early detection is critical, current non-invasive diagnostic tools have several limitations, including patients’ inability to exercise, contraindications for stress tests, radiation exposure, and high costs [1], [2]. Although advanced techniques such as CT-derived fractional flow reserve provide valuable functional information, their applicability remains limited in routine outpatient and preventive settings. Consequently, there is a clear clinical need for a simple, safe, and rapid screening tool suitable for early risk assessment [3], [4].

The Coronyzer (KH-3000) was developed to address this unmet need as a non-invasive pulse wave analysis device. It derives indices related to coronary resistance (R) and compliance (C) by integrating synchronized arterial pulse waveforms, electrocardiography, and blood pressure measurements. Rather than directly measuring absolute coronary pressure or flow, the device analyzes waveform morphology, timing, and area-based features referenced to central arterial hemodynamics, which have been shown to be more closely associated with coronary pathology than peripheral pressure measurements alone [5], [6], [7], [8], [9]. Through this signal integration framework using the modified Frank and Donald formula, Coronyzer provides non-invasive indices intended to reflect hemodynamic alterations associated with coronary artery disease, without requiring invasive catheterization or contrast agents [10], [11], [12].

The present study aimed to evaluate the diagnostic performance of Coronyzer in a prospective cohort of patients undergoing coronary angiography and to assess the robustness of its performance in an independent validation cohort. Importantly, the device estimates non-invasive surrogate indices of coronary hemodynamics rather than directly measuring invasive physiological parameters such as fractional flow reserve. Accordingly, Coronyzer is positioned as a screening and risk stratification tool designed to support clinical triage and referral for further diagnostic evaluation, rather than as a definitive physiological or therapeutic decision-making instrument.

## 2 Materials and methods

### 2.1 Device principle of the pulse wave analyzer Coronyzer

The Coronyzer derives coronary resistance and compliance from synthesized aortic pressure curves generated using carotid and femoral pulse waveforms, brachial blood pressure, electrocardiography, and phonocardiography in the Supplementary materials S1 [6], [7]. Mathematical modeling based on fluid dynamics principles was applied to estimate the coronary blood flow parameters [7], [13]. Detailed derivations are provided in the Supplementary Materials S2 and S3.

**Fig. 1:**
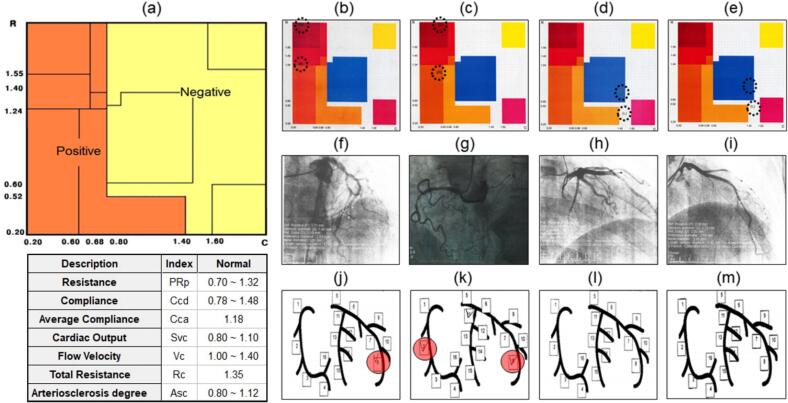
Efficacy evaluation of Coronyzer (KH-3000) for diagnosis of coronary artery disease in patients with angina pectoris. (a) C-R chart for the evaluation and diagnosis of myocardial infarction and coronary artery sclerosis coronary artery status. The orange area is a high probability area, indicating positive, and the yellow area is a low probability area, indicating negative. There are noticed seven coronary evaluation values of indices (Resistance PRp; Compliance Ccd, Average Compliance Cca, Cardiac Output Svc, Flow Velocity Vc, Total Resistance Rc, and Arteriosclerosis Asc) in the normal state (centered blue color box). (b–e) The results of diagnosis using the Coronyzer equipment for four patients are indicated with a double dotted line on the C-R chart. (f–i) Angiography diagnostic photos based on angiography results. (j–m) Lesion location through CAG for four patients as noticed in Table 2. (For interpretation of the references to color in this figure legend, the reader is referred to the web version of this article.)

C-R chart logic with analysis of result output involves calculating the bio-dynamic indicators from the area of the synthesized aortic arch internal pressure curve and displaying the results of cardiovascular analysis; calculating the S_l_ and S_r_ from the base of the left and right coronary arteries from the basic material including the area of the aortic arch internal pressure curve; calculating the C_l_ and C_r_, R_l_, and R_r_ by using the aortic arch internal pressure of the left and right coronary arteries by using the aortic arch internal pressure curve; and transmitting the results of cardiovascular analysis to the output for displaying one C-R chart with the calculated C_l_ and C_r_, R_l_, and R_r_ [6]. C_l_ and C_r_, R_l_, and R_r_ are shown as two points on the C-R chart of the analysis result display window of the output, as shown in Fig. 1(a).

#### 2.1.1 Area red zone (left and low) as area of stenosis

A darker red color indicates a higher probability of angiographic stenosis (per the device output), not a direct measure of stenosis severity. The darkest red region indicates an estimated >95% probability of stenosis by the device algorithm. The light ocher region indicates an estimated ∼70% probability; however, device probability does not reliably grade stenosis severity, and confirmatory testing is warranted when clinically indicated.

#### 2.1.2 Blue zone

Results in the blue region are considered test-negative; borderline results near the red zone should be interpreted cautiously and in conjunction with symptoms and pre-test probability. For a conservative interpretation, we considered compliance values ≥0.9 as more confidently test-negative (see threshold definitions below). In cases of discordance between the device and CAG, we classified disease status by the reference standard (CAG) and explored possible explanations (e.g., microvascular dysfunction) in the Discussion.

#### 2.1.3 Lower-right region

Interpretation depends on symptoms; patients with persistent or high-risk features should undergo further evaluation. This region may be observed in individuals with high cardiopulmonary fitness (e.g., athletes) and may be interpreted as normal in an appropriate clinical context.

#### 2.1.4 Pre-specified thresholds (R > 1.24 and/or C < 0.8)

The thresholds (R > 1.24 and C < 0.8) were pre-specified based on initial modeling and pilot data, and were not derived from receiver operating characteristic (ROC)-based optimization in the present cohorts [6], [14]. To avoid data-driven bias, thresholds were pre-specified based on modeling and pilot work rather than derived from the prospective cohort [2]. For evaluation, the C-R chart shown in Fig. 1(a) was divided into positive, high probability, low probability, and negative zones. The first two zones were defined as test-positive (suggesting CAD), and the last two zones were defined as test-negative (suggesting no significant CAD).

Resistance values are expressed in mmHg·min/L (equivalent to dyn·s/cm^5^ in CGS units), and compliance is expressed in mL/mmHg. Values are derived using a geometry-informed reference database incorporating age, sex, height, and weight parameters. The reported indices represent normalized surrogate measures rather than direct anatomical measurements.

### 2.2 Prospective study design

This multicenter prospective diagnostic accuracy study (IRB No. 3-2009-0118) was conducted at Yonsei University Gangnam Severance Hospital and Konyang University Hospital between January and September 2010. We enrolled patients aged 30–70 years referred for evaluation of suspected angina (Table 1). The exclusion criteria were prior coronary revascularization, acute myocardial infarction, severe heart failure, or contraindications to angiography.

**Table 1:** Baseline characteristics of two independent clinical study.

Variable	Initial prospective study (*n* = 94)	Retrospective validation study (*n* = 136)	p-Value
Age at prescription, years	57.9 ± 8.4	73.0 ± 9.5	<0.001
BMI, kg/m^2^	25.4 ± 3.3	24.4 ± 3.5	0.029
Sex			0.019
Male	61 (64.9%)	67 (49.3%)	
Female	33 (35.1%)	69 (50.7%)	
Hypertension	62 (66.0%)	96 (70.6%)	0.457
Diabetes mellitus	25 (26.6%)	35 (25.7%)	0.884
Dyslipidemia	65 (69.1%)	107 (78.7%)	0.102
Smoking status			0.045
Never smoker	62 (66.0%)	101 (74.3%)	
Ex-smoker	14 (14.9%)	24 (17.6%)	
Current smoker	18 (19.1%)	11 (8.1%)	
Previous history of CVD	29 (30.9%)	42 (30.9%)	0.996

Data are presented as mean ± standard deviation or number (percentage). BMI indicates body mass index; CVD, cardiovascular and cerebrovascular disease. P-values are provided for descriptive purposes only and were not used for inferential comparison between cohorts.

**Table 2:** Clinical data analysis of the four representative cases in Fig. 1(b), (c), (d), and (e).

Age	Sex	R_Left_	C_Left_	R_Right_	C_Right_	Asc_Left_	Asc_Right_	C-R chart	CAG	Lesion location	Decision
69	M	2.035	0.358	1.263	0.347	1.288	1.22	Fig. 1(b)	Fig. 1(f)	Fig. 1(j)	Positive
66	F	2.889	0.558	1.113	0.541	1.079	1.032	Fig. 1(c)	Fig. 1(g)	Fig. 1(k)	Positive
49	M	0.407	1.489	0.781	1.444	0.864	0.94	Fig. 1(d)	Fig. 1(h)	Fig. 1(l)	No CAD (by QCA)
38	F	0.492	1.478	0.888	1.434	0.901	0.979	Fig. 1(e)	Fig. 1(i)	Fig. 1(m)	No CAD (by QCA)

Quantitative coronary angiography was evaluated in a blinded manner by an independent specialist. The percentage-diameter stenosis was assessed using the lesion count from each coronary-artery surgery-study (CASS) dataset. For participants who elected interventional therapy (*n* = 94), stenosis severity was derived from continuous quantitative analysis performed with computerized quantitative angiographic (QCA) software (Pie-Medical, CASS-5, The Netherlands). Significant stenosis was operationally defined by a threshold of ≥ 50% diameter reduction.

All participants underwent Coronyzer KH-3000 testing before coronary angiography. Operators and angiography interpreters were blinded to the device results. Detailed clinical study protocols are provided in the Supplementary Material (S4). Coronyzer results were automatically generated and recorded before angiography, and angiography readers were blinded to Coronyzer findings. Likewise, device operators were unaware of angiographic results at the time of testing.

### 2.3 Validation study

An independent retrospective cohort comprising 136 patients who had undergone coronary angiography and coronary CT angiography between 2021 and 2025 was subjected to analysis (Table 1). Baseline characteristics were compared descriptively between cohorts. Continuous variables were compared using Welch's *t*-test, and categorical variables were compared using the chi-square test (2 × 2 or 2 × 3 as appropriate). *P*-values are provided to illustrate differences between cohorts for descriptive purposes only and were not for inferential comparison. A vessel-based assessment of the left and right coronary arteries was conducted. Given that two vessels per patient were evaluated, cluster-robust confidence intervals (or generalized estimating equations) were applied to accommodate within-patient correlation.

Diagnostic Rules as follows:

Two predefined diagnostic rules were evaluated:
  • OR rule: *R* > 1.24, or C < 0.8
  • AND rule: R > 1.24, and C < 0.8.

Using thresholds without recalibration were pre-specified on the basis of pilot studies and modeling to mitigate potential data-driven bias. The thresholds (R > 1.24 and C < 0.8) were derived from prior fluid-dynamic modeling and pilot observations before initiation of the prospective cohort and were not optimized using the present dataset, thereby minimizing the risk of overfitting [6], [14].

### 2.4 Statistical analysis method and number of subjects

The test results are organized into a 2 × 2 table and sensitivity and specificity are calculated. We assessed whether sensitivity and specificity exceeded pre-specified performance targets of 65% and 75%, respectively. At a 95% confidence interval, the margin of error, the allowable error, was set to 0.1, and considering both 0.65 (sensitivity) and 0.75 (specificity), the number of subjects was set to 100, which is sufficient. ROC analysis was performed to evaluate overall discriminatory performance and to visualize predefined operating points, rather than to derive optimal cut-off values. Confidence intervals for sensitivity, specificity, PPV, and NPV were calculated using the exact Clopper–Pearson method.

## 3 Results

### 3.1 Effectiveness of initial evaluation results

#### 3.1.1 Primary effectiveness evaluation items

The prevalence of ≥50% angiographic stenosis was 40.4% in the prospective cohort (38/94: ITT), was 34.6% in the validation cohort (47/136). Patients with ≥50% diameter stenosis on coronary angiography were classified as disease-positive; those with <50% stenosis were classified as disease-negative. For multivessel disease, we defined patient-level disease status using the most severe lesion (maximum percent stenosis). Agreement between the index test and reference standard was classified as true positive/true negative, and disagreement as false positive/false negative [Supplementary Material S4]. We compared the positive (+) and negative (−) results on the Coronyzer chart (Fig. 1(a)) and the cardiovascular angiography photos, as shown in Fig. 1(b–e) and (f–i), respectively. Clinical data analysis in Table 1 shows lesion location through coronary angiogram for four representative patients, as shown in Fig. 1(j–m). These different results were used to analyze the sensitivity and specificity of the primary effectiveness evaluation. If a point fell near a zone boundary, we applied the numerical R and C values to determine classification.

#### 3.1.2 Effectiveness evaluation results

This was a single-arm diagnostic accuracy study enrolling consecutive patients; there was no separate control group. A total of 94 patients were tested after excluding those who did not meet the inclusion criteria.

a. ***ITT (intention-to-treat) target group result analysis:*** The results of 100 participants who completed the clinical tests were analyzed. The comparison standards had a sensitivity of 65% and a specificity of 75%. Here, the test statistic is $z=\frac{\hat{p}-p}{\sqrt{\frac{p\left(1-p\right)}{n}}}$, $\hat{p}$ is the sensitivity (or specificity) obtained from the sample, and p is the value set for the null hypothesis. The rejection zone used a two-sided test; therefore, a critical value of 1.965 was used for the rejection and acceptance zones. In the ITT population, sensitivity was 81.4% (35/43; 95% CI, 0.666–0.916), and specificity was 87.7% (50/57; 95% CI, 0.763–0.949), as shown in Table 3. The test statistic was *z* = 2.256 at a sensitivity of 0.814 and *z* = 2.213 at a specificity of 0.877. As this satisfies the critical value of 1.965, the null hypothesis can be rejected.

**Table 3:** Diagnostic performance of Coronyzer vs. coronary angiography (ITT & PP).

Analysis	TP	FP	FN	TN	Sensitivity, % (95% CI)	Specificity, % (95% CI)	PPV, % (95% CI)	NPV, % (95% CI)	Accuracy, %
ITT (n = 100)	35	7	8	50	81.4 (0.666–0.916)	87.7 (0.763–0.949)	83.3 (0.686–0.930)	86.2 (0.746–0.939)	85.0
PP (n = 94)	31	6	7	50	81.6 (0.657–0.923)	89.3 (0.781–0.960)	83.8 (0.680–0.938)	87.7 (0.763–0.949)	86.2

TP: true positive, FP: false positive, FN: false negative, TN: true negative, PPV: positive predictive value, NPV: negative predictive value, CI: confidence intervals.
b. ***PP (per-protocol) target group result analysis:*** Out of 94 people, 74 were tested at Konyang University, and 26 people were tested at Yonsei University. Five of the 26 people tested at Yonsei University and one of the 74 people tested at Konyang University were excluded from the selection criteria and were therefore treated as missing, as shown in Table 3. The measurement failure rate was reported to be 6% (6 in 100) and clinical examination schedule was shown in Table S1 in Supplementary Material S4A. We analyzed the results of 94 individuals who met the selection and examination criteria and completed Coronyzer and cardiovascular angiography without dropping out. As this corresponds to a minimum sample size of ≥88, which satisfies both the sensitivity and specificity specified in the clinical protocol, the results are statistically significant. The standard for comparison was a sensitivity of 65% and a specificity of 75%, and a two-sided test was performed with a significance level of α = 0.05. In the PP population, sensitivity was 81.6% (31/38; 95% CI, 0.657–0.923), and specificity was 89.3% (50/56; 95% CI, 0.781–0.960), and the accuracy was 86.2% (Table 3). The positive predictive value (PPV) was 83.78%, and negative predictive value (NPV) was 87.72%. This result shows that regardless of the main analysis target group, the null hypothesis can be rejected for both the PP and ITT target groups, and there is almost no difference in the sensitivity and specificity results depending on the target group.

### 3.2 The validation evaluation results for 136 patients of retrospective dataset

A comparative analysis was performed using another existing registry study (Establishment of a cardiovascular geometric shape database, Yonsei University Gangnam Severance Hospital, IRB No. 3-2021-0133). All the data with simultaneously existing CAG are shown in Table 4. Computed tomography coronary angiogram (CTCA) and Coronyzer data were extracted during recent 5 years. We retrospectively identified 136 patients who underwent scheduled CAG and CTCA.

**Table 4:** Diagnostic performance in the validation cohort (OR vs AND rules).

Rule	TP	FP	FN	TN	Sensitivity, % (95% CI)	Specificity, % (95% CI)	PPV, % (95% CI)	NPV, % (95% CI)	Accuracy, %
OR	36	53	11	36	76.6 (0.620–0.877)	40.4 (0.302–0.514)	40.4 (0.302–0.514)	76.6 (0.620–0.877)	52.9
AND	25	20	22	69	53.2 (0.381–0.679)	77.5 (0.674–0.857)	55.6 (0.400–0.704)	75.8 (0.657–0.842)	69.1

TP: true positive, FP: false positive, FN: false negative, TN: true negative, PPV: positive predictive value, NPV: negative predictive value, CI: confidence intervals.

**Fig. 2:**
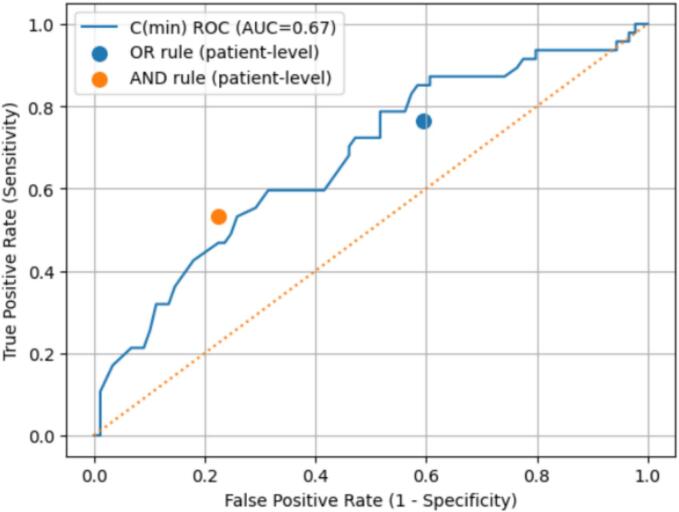
ROC curve (blue solid line) of false positive rate (FPR) vs. true positive rate (TPR) for area under the ROC curve (AUC = 0.67). Here, when the R and C values were calculated using OR rule, they were indicated with blue circle dot, and when calculated using AND rule, they were indicated with red circle dot. (For interpretation of the references to color in this figure legend, the reader is referred to the web version of this article.)

The diagnostic consistent performance of the Coronyzer device was evaluated using R and C indices under two predefined diagnostic rules: an OR rule and an AND rule, as shown in Fig. 2. When the OR diagnostic rule was applied (*R* > 1.24 or C < 0.8), Coronyzer demonstrated a high sensitivity of 0.77, with a specificity of 0.41, resulting in an overall accuracy of 0.53. Confusion matrix analysis (Table 4) showed a relatively low false-negative rate, indicating that most vessels with significant stenosis were correctly identified. However, the false-positive rate remains substantial, reflecting the limited specificity. These findings indicate that the OR rule prioritizes sensitivity over specificity, positioning the Coronyzer as an effective screening tool for detecting significant coronary artery stenosis.

Under the stricter AND diagnostic rule (*R* > 1.24 and C < 0.8), the sensitivity decreased to 0.53, while the specificity markedly increased to 0.78, with an overall accuracy of 0.69. This rule substantially reduces false-positive classifications at the expense of missing a proportion of stenotic vessels. Fig. 2 shows properties included of the ROC curve (blue solid line) of false positive rate (FPR) vs. true positive rate (TPR) for area under the ROC curve (AUC) = 0.67. Therefore, the AND rule demonstrated a strong rule-in capability, supporting its use as a confirmatory strategy following initial screening rather than as a standalone diagnostic test. A direct comparison of the two diagnostic rules revealed a clear trade-off between sensitivity and specificity. The OR rule consistently achieved a high sensitivity and limited specificity, whereas the AND rule achieved a high specificity and reduced sensitivity.

## 4 Discussion

This study demonstrates that Coronyzer provides clinically meaningful diagnostic information for early CAD screening. Instead of functioning as a definitive diagnostic tool, this device offers a flexible diagnostic strategy that can be adapted to different clinical needs. The OR rule prioritizes sensitivity and is well suited for initial screening, where minimizing missed disease is critical, whereas the AND rule emphasizes specificity and may support referral decisions for further invasive or advanced diagnostic evaluation. Importantly, these decision rules were predefined based on clinical strategy rather than data-driven optimization, thereby reducing the risk of overfitting. The moderate diagnostic accuracy observed is consistent with the device's focus on non-invasive hemodynamic surrogate indices, which may be altered in early or functional disease even in the absence of angiographically significant stenosis. This characteristic supports its role in early risk stratification.

In the prospective cohort, Coronyzer results were evaluated against coronary angiography according to a pre-specified clinical protocol. Diagnostic performance demonstrated high sensitivity (81%) and specificity (89%), and no adverse events were observed during testing. The short acquisition and analysis time highlights the procedural simplicity of the system. Unlike conventional non-invasive tests that are limited by exercise requirements, radiation exposure, or contrast use, Coronyzer is designed to address practical barriers encountered in routine outpatient and preventive settings. Accordingly, the device may complement existing diagnostic pathways by facilitating early risk assessment, particularly in patients for whom standard testing is not feasible.

The non-invasive indices derived by Coronyzer should be interpreted as surrogate markers reflecting integrated vascular and flow-related properties rather than as direct measurements of coronary physiology. Alterations in resistance and compliance may arise from a combination of epicardial atherosclerosis, early functional changes, and microvascular dysfunction. This framework provides a plausible explanation for the observed trade-off between sensitivity and specificity, as well as for false-positive findings when anatomical stenosis alone is used as the reference standard. Conversely, the AND rule mitigates such effects by requiring concurrent abnormalities in both indices, thereby improving specificity. Although invasive physiological indices such as fractional flow reserve were not available in the present study, the dual-rule strategy conceptually parallels rule-out and rule-in approaches commonly used in physiology-based diagnostics.

The retrospective validation cohort provides additional insight into real-world applicability. Although a validation comparison study using recent data is a retrospective study with moderate diagnostic power, the results were derived from real-world clinical data in routine clinical practice. These findings suggest that Coronyzer may be used as an initial screening test for patients with chest pain, implying diagnostic value comparable to that of existing non-invasive functional diagnostic tools. This inference is based on the fact that among patients who presented with chest pain, those who were clearly normal, those with evident myocardial infarction, or those with overt unstable angina were excluded; only patients with uncertain diagnoses were included. What distinguishes this work from earlier clinical trials is that, because the study reflected actual clinical use, it incorporated a substantial number of elderly patients or patients with lower limb problems who typically have difficulty undergoing exercise stress testing. This inclusion may represent an advantage for these patient populations. Because the cohort included a substantial proportion of elderly patients and individuals with limited ability to undergo exercise-based testing, the findings may be particularly relevant to populations that are often underrepresented in traditional stress-test studies. While the validation analysis was retrospective and of moderate size, the consistency of diagnostic profiles across cohorts supports the generalizability of the predefined decision rules. Although invasive physiological indices were not available, the dual-rule strategy mirrors the conceptual framework used in physiology-based diagnostics [15], [16], [17].

Several limitations should be acknowledged. First, this study relied on angiographic stenosis as the reference standard and did not incorporate invasive physiological measurements or outcome-based validation [18], [19]. Second, potential selection and spectrum biases, as well as confounding factors related to heart rate, blood pressure, and medication use, cannot be fully excluded. Third, reproducibility and longitudinal prognostic implications were not assessed. These limitations underscore the need for further investigation, and further validation across diverse ethnic and anthropometric populations is warranted, because coronary geometry is estimated using demographic parameters (age, sex, height, and weight). A key strength of this study is the vessel-based analysis and the use of alternative reference definitions, which demonstrate the stability of Coronyzer's diagnostic profile.

Future studies should aim to validate Coronyzer in larger, multicenter cohorts and to further refine diagnostic thresholds using independent datasets. Contextual comparison with established non-invasive functional modalities, such as CT-derived fractional flow reserve and quantitative flow ratio, may help clarify the device's role within diagnostic workflows. In addition, integration with complementary physiological approaches addressing both epicardial disease and coronary microvascular function may provide deeper insight into the pathophysiological meaning of the derived indices [15], [16], [17]. At present, Coronyzer might be regarded as a non-invasive screening and risk stratification tool rather than a definitive diagnostic or prognostic instrument, with particular utility in patient populations for whom conventional testing is limited, such as the elderly, patients who cannot exercise, and patients who have difficulty using contrast agents because of reduced renal function.

## 5 Conclusions

Coronyzer demonstrated clinically meaningful diagnostic performance as a rapid and safe non-invasive tool for the early screening and risk stratification of coronary artery disease. While the device is not intended to serve as a definitive diagnostic or prognostic instrument, it provides non-invasive surrogate indices that may support identification of patients who warrant further diagnostic evaluation. By avoiding radiation exposure, contrast agents, and exercise requirements, Coronyzer addresses several practical limitations of existing non-invasive testing modalities and may complement current diagnostic pathways.

Further validation in large-scale, multicenter studies is warranted to confirm the robustness and generalizability of these findings and to refine its clinical role. At present, Coronyzer may be particularly useful in elderly patients and in individuals unable to undergo exercise- or contrast-based testing, supporting its potential application in preventive cardiology and early risk assessment across diverse clinical settings, including both tertiary centers and smaller medical facilities.

## CRediT authorship contribution statement

**Byoung-Kwon Lee:** Writing – review & editing, Visualization, Validation, Resources, Methodology, Formal analysis, Conceptualization. **Seog-San Hyeon:** Resources, Project administration, Funding acquisition, Conceptualization. **YouSik Hong:** Writing – review & editing, Visualization, Software, Methodology, Investigation, Formal analysis, Data curation. **Dae-Woong Choi:** Software, Resources, Methodology, Investigation, Formal analysis, Data curation. **Sang-Suk Lee:** Writing – review & editing, Writing – original draft, Visualization, Validation, Supervision, Resources, Project administration, Methodology, Investigation, Formal analysis, Data curation, Conceptualization.

## Ethics approval and consent to participate

The clinical tests were approved by the Human Ethics Committee (Institutional Review Board, IRB) of Yonsei University Gangnam Severance Hospital, Korea, according to the Human Experiment Regulations (IRB No. 3-2009-0118, IRB No. 3-2021-0133).

## Declaration of competing interest

The authors declare that they have no known competing financial interests or personal relationships that could have appeared to influence the work reported in this paper.

## Appendix A Supplementary data

Supplementary material Image 1
