# Supplementary material for: Non-invasive estimation of coronary resistance and compliance: Prospective diagnostic study vs. angiography
Source: Am Heart J Plus. 2026 Mar 18;64:100763. doi: 10.1016/j.ahjo.2026.100763 (PMC13049544; doi:10.1016/j.ahjo.2026.100763)
Supplement: Supplementary file 1 — Supplementary material [file mmc1.docx]

**Non-invasive Estimation of Coronary Resistance and Compliance: Prospective Diagnostic Study vs. Angiography**

**Supplementary Materials (S1, S2, S3, S4)**

Byoung Kwon Lee, MD, PhD, ^a^ Seog-San Hyeon, ^b^ YouSik Hong, PhD, ^c^ Dae-Woong Choi, ^b^ Sang-Suk Lee, PhD, ^c,*^

^a^ *Cardiovascular Center, Department of Internal Medicine, Gangnam Severance Hospital, College of Medicine, Yonsei University, Seoul, 06273, Republic of Korea*

^b^ *Technology Research Institute, Irumedi Co., Ltd., Goyang, Gyeonggi-do,* *10442, Republic of Korea*

^c^ *Department of Software, College of Engineering, Sangji University, Wonju, Gangwon-do, 26339, Republic of Korea*

^*^ Corresponding author at: Department of Software, Sangji University, Wonju, Gangwon-do, 26339, South Korea*. E-mail address:* sslee@sangji.ac.kr ([S.](https://orcid.org/0000-0002-4706-180X) S. Lee).

**S1. Device operating principle and measurement method of the cardiovascular pulse wave analyzer Coronyzer (KH-3000)**

Fig. 1S shows five measurement methods and sites of sensor placement. Coronyzer (KH-3000) is equipped with a cuff-type mercury sphygmomanometer (Inbody Co. Ltd., Republic of Korea, Model BPBIO 210) near the brachial artery. The cardiovascular pulse waveforms are obtained by using two arterial pulse gram (APG) sensors (IRUMEDI Inc., Republic of Korea, Model IRKH 10.01.25-002) at the left and right cervical and femoral arteries. A heart sound phonocardiogram (PCG) sensor (IRUMEDI Inc., Republic of Korea, Model IRKH 10.01.25-003) and a wrist clip-type electrode for electrocardiogram (ECG) sensor (IRUMEDI Inc., Republic of Korea, Model IRKH 10.01.25-004) are attached near the heart and to left wrist, respectively. This cardiovascular pulse wave analyzer suggests reasonable treatment methods based on the diagnostic results and further enables the analysis of the prognosis of the disease. Coronyzer could offer a new method for the medical diagnosis of heart diseases. Although this procedure is simple and easy, it examines the detailed condition of the coronary arteries.


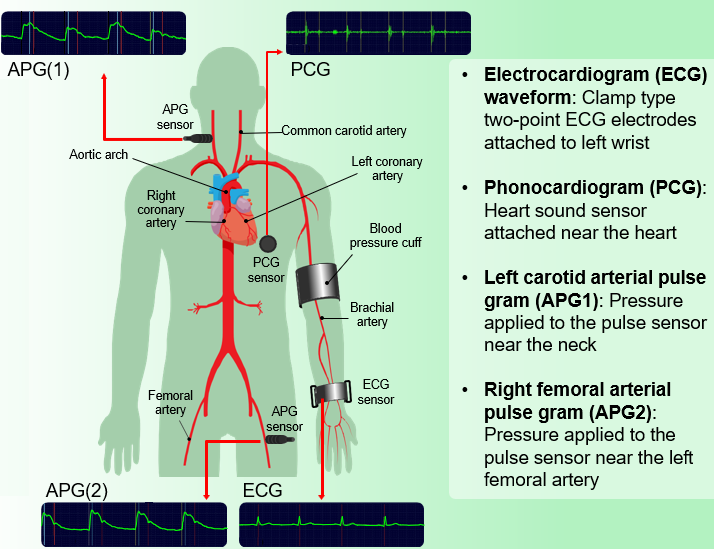


**Fig. 1S.** The schematic of measurement methods and sites of sensor placement for Coronyzer analyzer. The device includes three clamp-type electrodes for ECG measurement, two APG sensors for the carotid and femoral arteries, one heart sound PCG sensor, and one brachial artery cuff-type blood pressure monitor.

Fig. 2S shows a photograph of the actual measurement and a detailed each waveform using a sensor attached to Coronyzer and a separate tool. Firstly, the ECG waveform was measured by attaching a clamp type two-point electrode with ECG electrodes to the left wrist. Secondly, the PCG waveform shows the heart-sound sensor attached to the heart. Thirdly, Fig. 2S(b) shows the pulse waveform obtained by applying pressure to the APG sensor near the neck, where the left carotid artery is located. Additionally, a cuff-type blood pressure monitor was mounted on the forearm near the brachial artery. This represents an actual measurement of high blood pressure. Fig. 2S(c) shows the pulse waveform obtained by applying pressure to the APG sensor near the left femoral artery. The ECG, heart sound, and left/right carotid waveforms, measured using various measurement tools attached to Coronyzer described in Fig. 1S, were shown on the display screen of the analysis device.

When examining the ECG, PCG, and the left and right carotid APG waveforms measured simultaneously for approximately 5 s, the Q-point peak of the ECG waveform was the same as that of the diastolic pulse waveform. This is indicated by a line. In addition, the two peak band widths shown in the heart sound waveform are distinguished by a yellow line corresponding to the peak band width of the starting point and notch point of the diastolic pulse waveform. Fig. 2S(a) shows the arterial parts of the vascular system to help understand how to non-invasively measure pulse waves using Coronyzer [1s,2s]. In addition, the interval between the peak values ​​of the pulse waveform distinguished by the light-blue line is the same as the detailed division of the pulse waveform.

The two pulse waveforms measured using two APG sensors separately from the right and left carotid arteries. For the left carotid artery, as shown in Fig. 2S(b), a pulse waveform with distinct traveling and reflected waves appears in the form of an APG in Fig. 2S(b) because the arteries are close to the coronary artery and aorta. A detailed analysis of this process is presented in Fig. 2S(b); the period is *T* = 0.98 s, the pulse rate is 62.27, and the interval between the systolic and notch peaks is *T_s_* = 0.28 s. Meanwhile, as shown in Fig. 2S(c), the pulse waveform of the left femoral artery exhibits slightly reduced reflected waves compared to the traveling wave. This overlap appears in the coronary artery and aorta, as well as arteries slightly farther. The APG representation can be seen in Fig. 2S(c). If this is analyzed in detail, it appears as a value at the center of the bottom (i.e., the period is *T* = 1.08 s, the pulse rate is 72.29, the interval between the systolic peak and the notch peak is *T_s_* = 0.30 s, and the diastolic area is *A_d_* = 73.29).


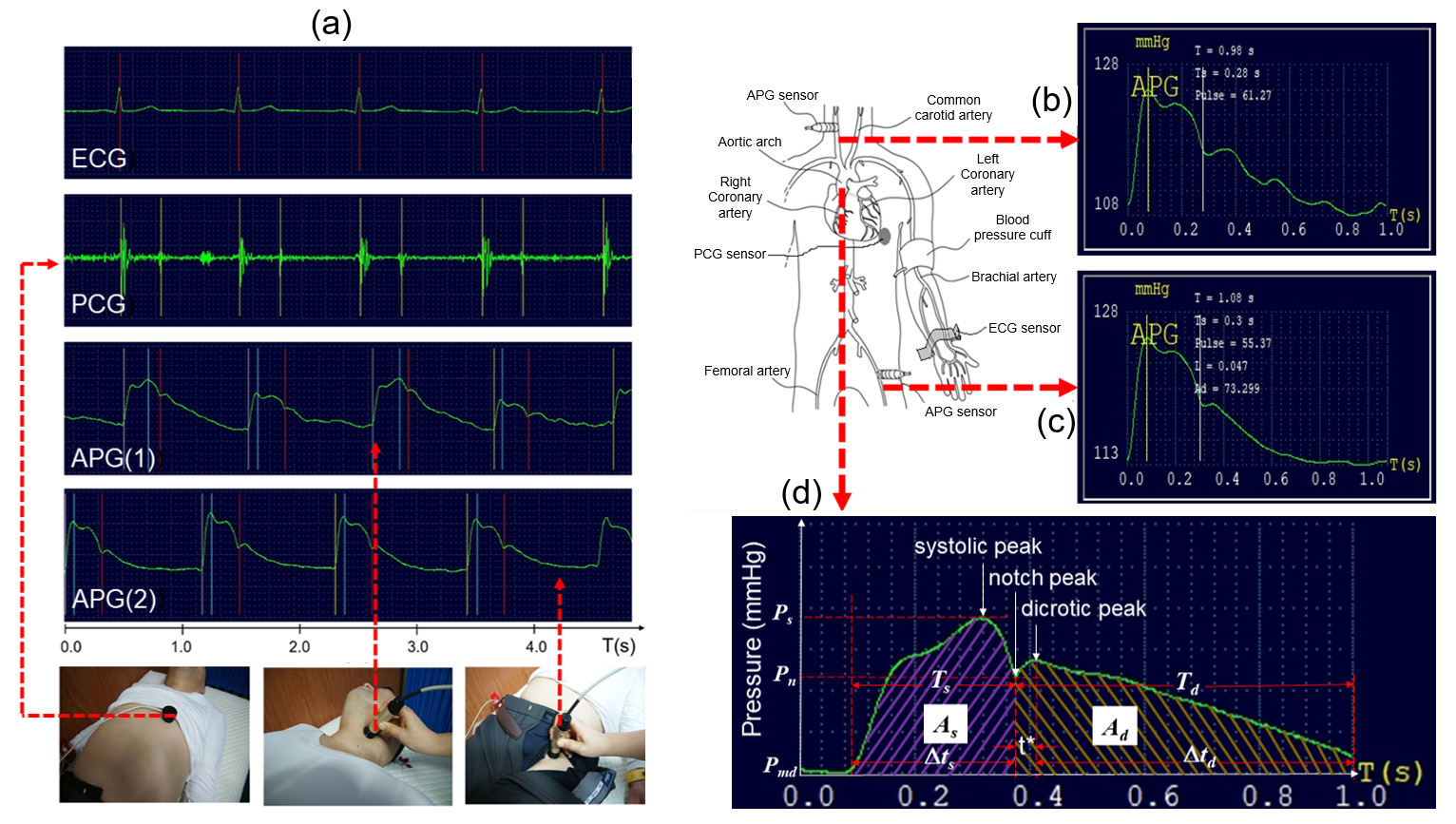


**Fig. 2S.** (a) The five measurement processes included: ECG, PCG, left carotid arterial pulse wave, and right femoral arterial pulse wave using two APG sensors. Diastolic signal analysis was performed for the waveforms of ECG, PCG, and APG in the diastolic pressure phase. Synthetic signal analysis was performed for (b) and (c) two waveforms of APG(1) and APG(2), respectively. (d) The main artery internal pressure curve inside the aorta driven by left and right carotid pulse waves in the diastolic and systolic states. Here, *T*, *T_s_*, *t*∗, ∆*t_s_*, ∆*t_d_*, *A_s_*, and *A_d_* are defined by period, notch peak time, dicrotic peak time, systolic interval time, diastolic interval time, systolic area, and diastolic area, respectively.

The internal pressure curve of the aortic arch, as shown in Fig. 2S(d), can be obtained from the two left and right carotid artery pulse waveforms measured during diastole, and the two pulse waveforms obtained from the left and right femoral arteries. The pulse waveform of Fig. 2S(d) shows the internal pressure curve of the aortic arch. Through this, key variables that are important in the formula for coronary blood flow and blood pressure can be defined, and the condition of the subject's coronary artery can be determined. Coronyzer creates a database of coronary artery length and diameter values ​​based on race, age, sex, height, and weight and creates an aortic arch pressure curve under the premise that the aortic arch and coronary artery inlet values ​​are identical according to Pascal's law. Blood flow in the coronary artery is calculated. The internal pressure curve of the aortic arch is obtained by combining the pulse waves of the left and right carotid arteries and the diastolic-systolic waveforms of the brachial cuff wave using a specially developed program. Based on coronary artery blood flow, the Hagen-Poiseuille and Windkessel equations were applied to measure blood flow resistance, compliance, and coronary artery stiffness. Because the resistance value expressing the obstruction of blood flow and compliance indicates the elasticity of blood vessels, the ischemic status is identified using these two indicators, and the coronary artery stiffness value determines stenosis [3s-6s].

**S2. The aortic arch derivation of the coronary artery blood flow formula obtained from the pressure curve**

Coronyzer, a cardiovascular pulse wave analyzer, is a new coronary artery evaluation model based on blood flow and blood pressure at the coronary artery inlet, which can be used as a diagnostic device for angina pectoris and myocardial infarction. According to Pascal’s law, the left and right coronary blood pressure inlet pressures are equal. The left and

right coronary blood flows can be determined by creating a coronary blood flow formula [7s]. The blood flow volume, *S_v_*, of the left coronary artery was calculated as follows: Kochoukos et al. applied Eq. (1) using the pressure pulse wave plots [8s,9s], where *T_s_* and *T_d_*, defined in Fig. 2S(d), are the systolic and diastolic durations, respectively, and A_s_ is the systolic area. *K* is also a constant that represents individual differences and can be confirmed by comparison with the cardiac output measured using standard methods.

$$S_{v}=K\cdot A_{s}\cdot\left[ 1+\frac{T_{s}}{T_{d}} \right] (1)$$

Kochoukos suggested that *K* can be expressed by Eq. (2) where *σ* is the Poisson’s coefficient (*σ* ≈ 0.5), *ρ* is the blood density (*ρ* = 1.05), and *PWV* is the wave velocity, which is the pulse wave transmission speed. The range of constant *k* obtained from a series of flow curves is 0.58–0.74 [10s].

$$K=k\cdot\frac{R^{2}}{PWV} \cdot\frac{({1-\sigma^{2})}^{1/2}}{\rho} (2)$$

Therefore, the amount of blood flow in the left coronary artery is *S*_L_, and *∆t_d_* in the above equation is the time from the endpoint of the overlapping wave to the point of dilation. *t*∗ is the time from the notch point to the expansion point.

$$S_{L}=k\cdot A_{d} \cdot\left( \frac{t^{*}+t_{d}}{\Delta t_{d}} \right) (3)$$

Blood flow in the right coronary artery can be obtained using Eq. (4). Here, *k*_1_ and *k*_2_ are constants obtained from the pressure and flow curves, respectively, and their range is 0.58–0.74. *σ* is Poisson's constant.

$$S_{R}=k_{1}\cdot{{(1-\sigma^{2})}^{1/2}\cdot P}_{md} \cdot\frac{\left( 1+\frac{A_{d}}{k_{2}\cdot A_{s}} \right)}{C} (4)$$

In addition, *A_s_* and *A_d_* are the systolic and diastolic pressure curves, respectively; *P_md_* is the average blood pressure; and *C* is compliance. The coronary artery system was modeled using two coronary arteries, and Eq. (3) and Eq. (4) are derived using this model. The formula obtained under this model is completely different from that for the central aorta and simply represents the coronary artery characteristics. A new concept that considers intratissue pressure was considered when modeling the coronary arteries. It is a new, unprecedented coronary artery evaluation model derived based on blood flow and blood pressure at the coronary artery inlet; therefore, it has no relation to the central aorta. The cardiovascular pulse wave analyzer Coronyzer KH-3000 determines whether the subject is currently in a state of ischemia (myocardial infarction in severe cases). If the patient is ischemic, it is possible to determine whether this ischemia is due to coronary artery stenosis, convulsions, or twitching. Cases of spasm without coronary artery stenosis are analyzed separately to determine whether it is a functional or an organic condition. Lifestyle intervention is possible by analyzing the chronically dominant state of the sympathetic nervous system.

**S3. The formulas and units of Coronary resistance (R), compliance (C), and atherosclerosis (Asc) derived from the coronary artery blood flow formula**

There are two types of blood flow resistance: peripheral resistance, which considers afterload, and blood flow resistance, which is based on the properties and elasticity of the blood vessel wall when the blood flows. The coronary artery has 500 branches, with 150 branches having a diameter of 0.5 mm or less. However, in the diastolic phase, intratissue pressure in blood vessels of 0.5 mm or less in the left coronary artery exists. Therefore, the peripheral resistance of the left coronary artery must be obtained, and the right coronary artery does not exhibit such a phenomenon. Accordingly, only blood flow resistance is calculated [11s,12s].

Because blood flow in the left coronary artery is caused by diastolic blood pressure, the peripheral resistance *R_L_*_1_ is calculated as follows:

$$R_{L1}=\frac{P_{d}-P_{v}}{S_{L}} (5)$$

where *P_d_* is the diastolic pressure, *P_v_* is the blood pressure measured at a random location, the venous pressure, and *S_L_* is the blood flow, which is the cardiac output of the left coronary artery.

In contrast, when blood flows into a blood vessel, the channel resistance *R_L_*_2_ generated in the channel of the blood vessel is given by the following equation:

$$R_{L2}=\frac{\bar{P_{d}}}{S_{L}} (6)$$

Here, $\bar{P_{d}}$is the diastolic mean blood pressure, and *S_L_* is the blood flow in the left coronary artery.

When blood flows through the peripheral resistance (*R_L_*_1_) of the left coronary artery and the channel resistance (*R_L_*_2_) of the left coronary artery, the blood flow resistance of the right coronary artery (*R_R_*), which is the blood flow resistance in the right coronary artery, is calculated as follows:

$$R_{R}=\frac{K_{2}A_{s}+A_{d}}{S_{R}} (7)$$

where *K*_2_ is a constant related to intratissue pressure, with a range of 0.7 to 0.75, *A_s_* is the area

Adaptability refers to the change in volume according to the change in unit pressure. In the equation below, compliance (*C_L_*) refers to the diastolic compliance.

$$C_{L}=\frac{{(S}_{L}- A_{d}/R_{L})}{(P_{n} - P_{d})} \left( 8 \right)$$

The above equation has been obtained from the expression on the previous page:

*S_L_*: Coronary blood flow

*A_d_*: Area of the diastolic aortic arch pressure curve

*R_L_*: Blood flow resistance in the left coronary artery

*P_n_*: Blood pressure at the notch

*P_d_*: Diastolic blood pressure

The right coronary artery is conformable considering the systolic and diastolic phases. That is, the compliance *C_R_* in the right coronary artery is calculated as follows: where *K*_2_*A_s_ - A_d_* is changed to *A_s_* in the case of the aorta but considering the case of the coronary artery. Adaptability is an index that indicates the extent to which a blood vessel expands when a unit force of a unit volume is applied to the blood vessel. Because the elasticity of a blood vessel is the same when the blood vessel is dilated or when the expanded blood vessel is contracted, conformability can be obtained as described above. Mechanically, this medium is referred to as an orthotropic medium.

$$C_{R}=\frac{K_{2}A_{s}- A_{d}}{P_{s} - P_{d}} \frac{S_{R}}{K_{2}A_{s}+ A_{d}} (9)$$

The degree of arteriosclerosis, *Asc_L_*, in the left coronary artery is given by the following equation:

$${Asc}_{L}=K_{3}\frac{{R_{L1}}^{0.25}}{C_{L}R_{L1}} (1+S) (10)$$

From the formula derived above, the following formulae are established.

$$C=\frac{A}{{(PWV)}^{2}} (11)$$

$$R=\frac{8}{A^{2}} (12)$$

where *ρ* and *η* are the density and viscosity of the blood, respectively. Meanwhile, according to the Moen-Korteweg equation, the pulse wave propagation velocity is $PWV= \sqrt{E\frac{h}{2r\rho}}$, where *h* is the wall thickness of the vessel, *r* is the inner diameter of the vessel, and *ρ* is the density of the blood [13s].

The modulus of elasticity (*E*) is calculated as follows:

$$E=\frac{2r}{h}{(PWV)}^{2} (13)$$

Substituting *PWV* in Eq. (10) with *C* in Eq. (11) and substituting *A* with *R*, the following equation is formed:

$$Asc=f(h)\frac{R^{0.25}}{CR} (14)$$

The change in the thickness of the artery according to the pulse-wave propagation velocity is calculated as follows:

$$f\left( h \right)=K_{3}[1+(PWV_{0}/{PWV)}^{0.7}] (15)$$

*K*_3_ is a coefficient obtained in clinical practice and ranges from 0.7 to 0.89. By analogy with the above formula, we can obtain the following formula:

$$Asc=K_{3}\frac{R^{0.25}}{CR}[1+(PWV_{0}/{PWV)}^{0.7}]=K_{3}\frac{R^{0.25}}{CR}(1+U) (16)$$

In the above formula, *U* = *f*(*PWV*) = (*PWV*_0_/*PWV*)^0.7^ is described. Because it is acceptable to give only the concept to the patent, the detailed official development is omitted.

Finally, the units for the three important measurement variables *C*, *R*, and *Asc* here are as follows: *R* is dyn·s/cm^5^ (CGS units) or mmHg·min/L, *C* is mL/mmHg and *Asc* is N/m^2^ [2s].

**References**

[1s] M. Zuber, M. Zellweger, J. Bremerich, CAd. Mauer, P. T. Buser, Non-invasive diagnostic of coronary artery disease, Therapeutische Umschau 66 (4) (2009) 241-251.

[2s] K. T. Kim, S. S. Hyeon, Cardiovascular analyzer, USA Patent No. US 8,771,195 B2, July 8 (2014).

[3s] J. Lee, N. P. Smith, The Multi-Scale Modelling of Coronary Blood Flow, Ann. Biomed Eng. 40 (2012) 2399–2413.

[4s] A. Fronek, R. W. Barnes, D. S. Sumne, Non-invasive Diagnosis in Vascular Disease, Appleton Davies, Pasadena, CA, USA. (1989).

[5s] S. P. Sutera, R. Skalak, The History of Poiseuille’s Law, Ann. Rev. Fluid Mech. 25 (1993) 1-20.

[6s] B. Lambermont, P. Gérard, O. Detry, P. Kolh et al., Comparison between three- and four-element Windkessel models to characterize vascular properties of pulmonary circulation, Arch. Physiol. Biochem, 105 (7) (1997) 625-632.

[7s] T. W. Seo, J. S. Byun, Computational Hemodynamics in the Intracranial Aneurysm Model, Trans. Korean Soc. Mech. Eng. B 37 (10) (2013) 927-932.

[8s] J. Lee, L. Lee, H. K. Lee, K. J. Lee, Sleep Apnea Detection using Estimated Stroke Volume, J. Biomed. Eng. Res. 34 (2) (2013) 97-103.

[9s] N. T. Kouchoukos, L. C. Sheppard, D. A. McDonald, Estimation of stroke volume in the dog by a pulse contour method, Circ. Res. 26 (5) (1970) 611-623.

[10s] S. W. Cho, S. Kim, K. C. Ro, H. S. Ryou, Study of Blood Characteristics in Stenosed Artery under Human Body Rotation by Using FSI Method, Trans. Korean Soc. Mech. Eng. B 37 (5) (2013) 449-457.

[11s] K. X. J. Zhang, K. Hoshino, Microfluidics and Micro Total Analytical Systems, In: *Molecular Sensors and Nanodevices*. Academic Press, Chap. 3 (2018) 43-111.

[12s] P. Rock, G. A. Patterson, S. Permutt, J. T. Sylvester, Nature and distribution of vascular resistance in hypoxic pig lungs, J. Appl. Physiol. 59 (6) (1985) 1891-1901.

[13s] B. H. Jin, M. H. Han, The Correlation of Pulse Wave Velocity and Atherosclerotic Risk Factor in Stroke Patients, Korean J. Clin. Lab. Sci. 47(1) (2015) 28-34.

**S4. Clinical examination protocol in first initial prospective clinical study**

**S4A. Clinical examination schedule in prospective diagnostic study**

**Table S1.** The typical result generation time and clinical examination schedule.

| Time (day) | Clinical Examination | Remark |
| --- | --- | --- |
| D-2, or D-7 | Patient’s visit and registration | Patients suspected of having coronary artery disease |
| D-1, or D-day | Hospitalization, ECG, echocardiography | Preliminary clinical examination, and complete informed consent form |
| D-day | Coronyzer (KH-3000) test | - |
| D-day, or D+1 | Coronary angiography (CAG) | CAG diagnosis and management |
| D+1, or D+2 | Comparison of test results | Comparative evaluation of CAG and Coronyzer results |

**S4B. Preparation for clinical trials of Coronyzer and blinding to angiography**

1. ***Patient Registration:*** This is a clinical trial of a medical device involving consecutive patients without a control group. Patients presenting with chest pain are enrolled in the outpatient clinic.
2. ***Operator training:*** If the subject is hospitalized in advance, the Coronyzer test is performed the afternoon before the cardiovascular angiography examination. If the subject is admitted in the morning, the Coronyzer test is performed simultaneously with the pre-examination, including ultrasound. The cardiovascular angiography is then performed in the angiography room.
3. ***Patient allocation:*** A total of 100 patients will be tested, with 25% to 75% allocated to each clinical testing center, depending on the circumstances, and the test will be conducted at Konyang University Hospital and Yonsei University Gangnam Hospital.
4. ***Blinding to angiography:*** The results of the Coronyzer for patients are not sent to the cardiovascular angiography room, and the physician performing the angiography is not made aware of the results of the Coronyzer. The results of the Coronyzer are automatically displayed in the area of ​​the interpretation sheet, so manual or arbitrary adjustments are impossible. In addition, the results of the cardiovascular angiography are interpreted and recorded on the spot after the angiography is performed, so no bias from the subject, examiner, or interpretation can intervene.

**S4C. C-R chart logic with analysis of result output**

1. Calculating the bio-dynamic indicators from the area of the synthesized aortic arch internal pressure curve and displaying the results of cardiovascular analysis
2. Calculating the S_l_ and S_r_ from the basic of left and right coronary arteries from the basic Material including the area of aortic arch internal pressure curve
3. Calculating the C_l_ and C_r_, R_l_ and R_r_ by using the aortic arch internal pressure of left and right coronary arteries by using the aortic arch internal pressure curve
4. Transmitting the results of cardiovascular analysis to the output for displaying one C-R chart with the calculated the C_l_ and C_r_, R_l_ and R_r_
5. Showing the C_l_ and C_r_, R_l_ and R_r_ as two points on the C-R chart of the analysis result display window of the output, as shown in Fig. 1(a).
6. ***Area red zone (left and low) as area of stenosis:*** A darker red color means a higher rate of stenosis is not severe stenosis. The reddest area means that there is a more than 95% probability of stenosis. Light ocher area means that there is 70% a probability of stenosis. But it may be severe stenosis, so the patient’s condition should be carefully monitored and further examination should be determined.
7. ***Area blue zone as area of normal:*** The border area close to the left (red zone) should be carefully monitored. It’s safer to say normal that C result should be 0.9 or higher. Other area is normal. Coronary artery condition is not very healthy, but the CAG is normal.
8. ***Area right low as observation depends on symptoms:*** If you have symptoms, a thorough examination is required. Most people with good cardiopulmonary functions, such as athletes, come out to this area and judge it as normal.
9. ***Thresholds for a positive decision (R > 1.24, C< 0.8):*** To avoid data leakage, the thresholds were not derived from the 94 patients who participated in this clinical trial, but were prespecified based on initial fluid dynamic modeling and pilot studies [2s].

**S4D. Efficacy and safety-related variables and quality assurance**

1. ***Statistical analysis method and number of subjects:*** The test results are organized into a 2×2 table and sensitivity and specificity are calculated. The results are non-inferior or superior to the expected sensitivity of 65% and specificity of 75%. At a 95% confidence interval, the margin of error, the allowable error, is set to 0.1, and considering both 0.65 (sensitivity) and 0.75 (specificity), the number of subjects is set to 100, which is sufficient.
2. ***Coronyzer diagnostic evaluation method:*** For evaluation, the C-R chart shown in Fig. 1(a) is divided into positive, high probability, low probability, and negative zones. The first two zones were defined as positive without disease, and the last two zones were defined as negative without disease.

# S4E. Confirmation-observation-analysis of adverse reactions during test of Coronyzer

**Table S2.** Cause analysis of Coronyzer test


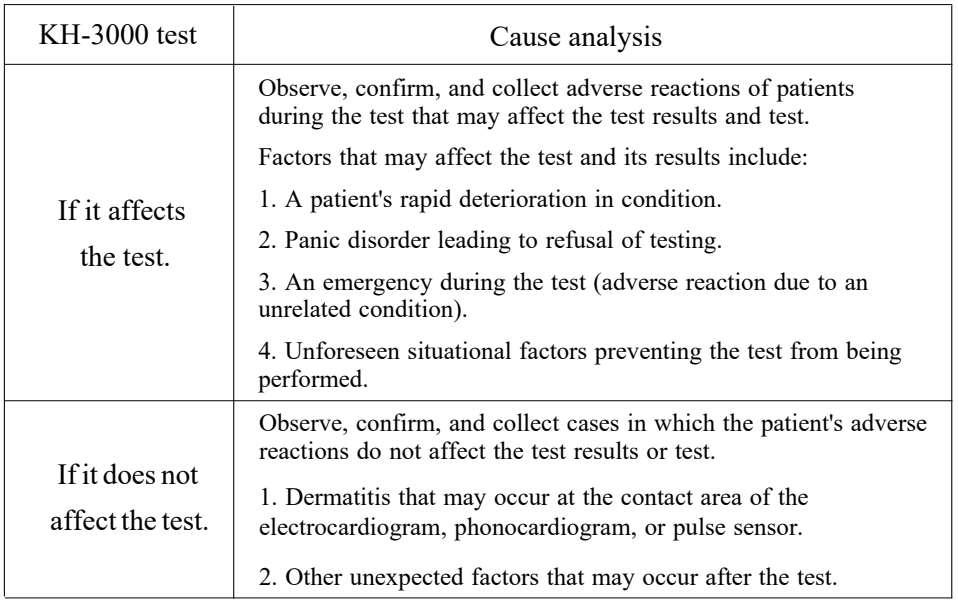


1. During the Coronyzer test, abnormal reactions are confirmed, observed, and collected, as shown in Table S2. Observe all adverse reactions that may occur during clinical trials of Coronyzer. If an adverse reaction is discovered, immediately identify it and record it in the adverse reaction section of the Case Report Form (CRF).
2. Analysis of adverse reactions: Adverse reactions are identified and analyzed individually through the CRF table and follow-up observations. Furthermore, for adverse reactions directly related to Coronyzer, the cause is analyzed and the incidence rate for each reaction is recorded. Each incidence rate is tabulated and reported to the clinical investigator, and attached to the clinical trial report.
